# Microbial enhanced oil recovery through deep profile control using a conditional bacterial cellulose-producing strain derived from *Enterobacter* sp. FY-07

**DOI:** 10.1186/s12934-020-01314-3

**Published:** 2020-03-05

**Authors:** Ge Gao, Kaihua Ji, Yibo Zhang, Xiaoli Liu, Xuecheng Dai, Bo Zhi, Yiyan Cao, Dan Liu, Mengmeng Wu, Guoqiang Li, Ting Ma

**Affiliations:** 1 Key Laboratory of Molecular Microbiology and Technology, Ministry of Education, College of Life Sciences, Nankai University, Tianjin, 300071 People’s Republic of China; 2 Tianjin Key Laboratory of Radiation Medicine and Molecular Nuclear Medicine, Department of Radiobiology, Institute of Radiation Medicine of Chinese Academy of Medical Science & Peking Union Medical College, Tianjin, 300192 People’s Republic of China; 3 Research Institute of Experiment and Detection, Xinjiang Oilfield Branch Company, PetroChina, Karamay, 834000 Xinjiang People’s Republic of China

**Keywords:** Microbial enhanced oil recovery, Deep profile control, Bacterial cellulose, Heterogeneity, Premature plugging

## Abstract

**Background:**

Heterogeneity of oil-bearing formations is one of major contributors to low oil recovery efficiency globally. Long-term water flooding will aggravate this heterogeneity by resulting in many large channels during the exploitation process. Thus, injected water quickly flows through these large channels rather than oil-bearing areas, which ultimately leads to low oil recovery. This problem can be solved by profile control using polymer plugging. However, non-deep profile control caused by premature plugging is the main challenge. Here, a conditional bacterial cellulose-producing strain, namely *Enterobacter* sp. FY-0701, was constructed for deep profile control to solve the problem of premature plugging. Its deep profile control and oil displacement capabilities were subsequently identified and assessed.

**Results:**

The conditional bacterial cellulose-producing strain *Enterobacter* sp. FY-0701 was constructed by knocking out a copy of fructose-1, 6-bisphosphatase (FBP) encoding gene in *Enterobacter* sp. FY-07. Scanning electron microscope observation showed this strain produced bacterial cellulose using glucose rather than glycerol as the sole carbon source. Bacterial concentration and cellulose production at different locations in core experiments indicated that the plugging position of FY-0701 was deeper than that of FY-07. Moreover, enhanced oil recovery by FY-0701 was 12.09%, being 3.86% higher than that by FY-07 in the subsequent water flooding process.

**Conclusions:**

To our knowledge, this is the first report of conditional biopolymer-producing strains used in microbial enhance oil recovery (MEOR). Our results demonstrated that the conditional bacterial cellulose-producing strain can in situ produce biopolymer far from injection wells and plugs large channels, which increased the sweep volume of injection water and enhance oil recovery. The construction of this strain provides an alternative strategy for using biopolymers in MEOR.

## Background

More than two-thirds of crude oil continues to remain in reservoirs after primary and secondary oil recovery [1–3], which is mainly attributable to the heterogeneity of reservoirs globally [4, 5]. During the water flooding process, injected water quickly flows through high permeability channels to produce wells, leading to recovery of a small fraction of crude oil [6, 7]. To tackle this problem, polymers are usually used to selectively plug high permeability zones, change the direction of water flow, modify the profile, improve volumetric sweep efficiency, and ultimately enhance oil recovery [8–10].

Polyacrylamide is currently the most commonly used polymer in laboratory and field trials [11–13]; however, as it is a chemical polymer, it cannot be easily biodegraded and is thus toxic to the environment [14]. Many non-toxic biopolymers, such as xanthan gum, can reportedly be efficiently used for selective plugging and profile control [15–17], but xanthan gum, for example, is a water-soluble biopolymer and is thus extremely sensitive to biodegradation [18, 19]. Therefore, it is imperative to identify cheap, stable, non-toxic alternatives. Bacterial cellulose (BC), a water-insoluble polysaccharide, is non-toxic and has a long degradation period [20]. *Enterobacter* sp. FY-07 is a Gram-negative bacterium with a fast growth rate and high salt tolerance; it can produce BC at 24–39 °C under different oxygen conditions using economic sources of carbon such as molasses and crude glycerol [21–23]. Hence, FY-07 shows great potential for selective plugging and profile control in order to enhance oil recovery. Moreover, considering the absence of oxygen in deep reservoirs, this facultative anaerobe is a good candidate for deep profile control [24].

Deep profile control is essential because in extensively developed reservoirs, residual oil is still present in zones far from the injection well [7]. The sweep volume and enhanced oil recovery (EOR) are positively correlated with profile control depth [25, 26]. BC is a primary metabolite of FY-07; FY-07 growth is accompanied by BC production, which, however, is not beneficial for FY-07 migration. On injecting FY-07 into the well, it tends to plug the supply pipes and walls of the well bore instead of high permeability zones [16, 27]. This makes it difficult to perform deep profile control with FY-07. To deal with this issue, a feasible strategy is to degrade BC when preparing bacterial cells for injection, but this requires special equipment and the use of cellulase, which makes the process expensive. Another feasible strategy is to use FY-07 for constructing a conditional BC-producing strain so as to avoid the production of BC during the injection process and to synthesize BC for deep profile control only upon reaching the deep areas of reservoirs.

The objective of this study is to obtain a conditional bacterial cellulose-producing strain for deep profile control in EOR. In this study, we derived a genetically engineered strain, *Enterobacter* sp. FY-0701, using the gene knockout approach. This strain could use glucose rather than glycerol as the sole carbon source to produce BC. BC production by FY-0701 was observed using scanning electron microscopy (SEM); moreover, the deep profile control ability of this strain was investigated using core flooding experiments and SEM, and EOR was verified using oil displacement experiments. Overall, the genetically engineered stain is promising for deep profile control in EOR.

## Results and discussion

### Determination of target genes for constructing conditional BC-producing strain

**Fig. 1:**
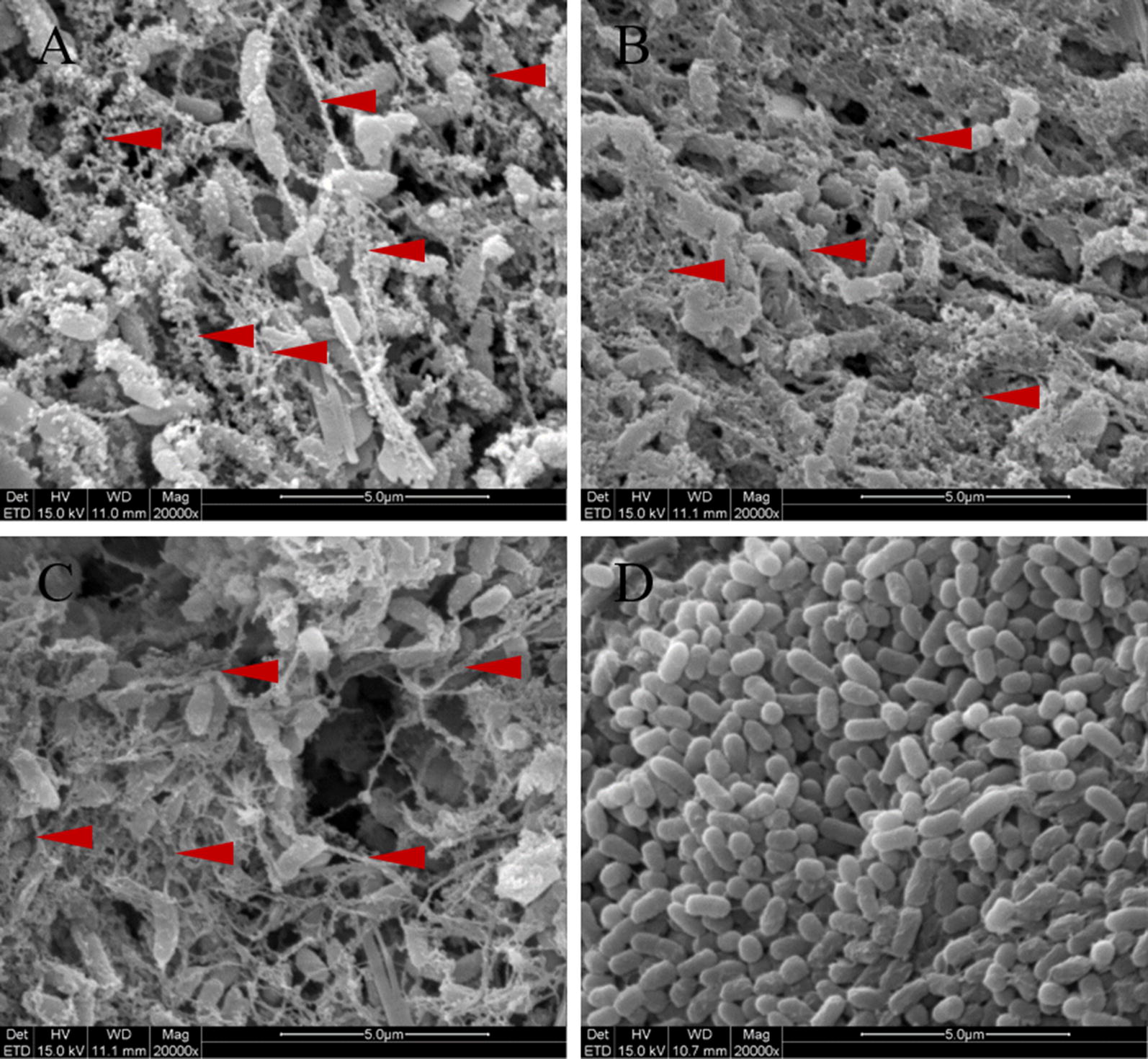
BC production comparison. *Enterobacter* sp. FY-07 and FY-0701 were streaked on fermentation media containing glucose (**a** and **c**) or glycerol (**b** and **d**) as the sole carbon source. Colonies of FY-07 (**a** and **b**) or FY-0701 (**c** and **d**) were selected to detect BC production by SEM observation. The magnification is 20,000×. Red triangles indicate parts of BC microfiber. *BC* bacterial cellulose

**Fig. 2:**
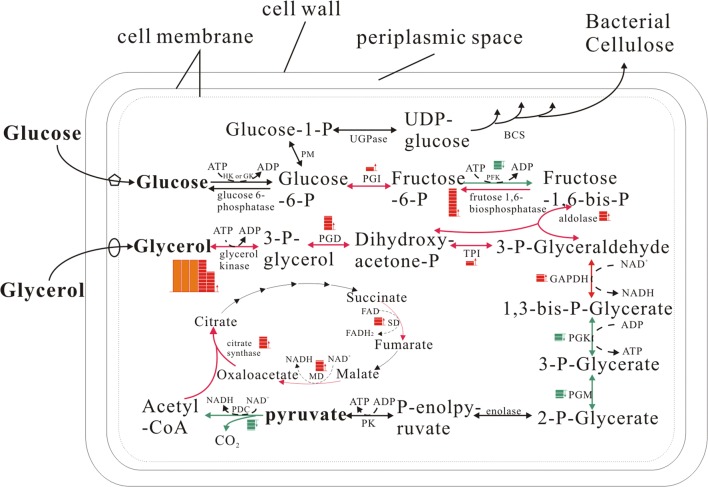
Changes in the transcription levels of genes related to glycolysis, gluconeogenesis, and glycerol metabolism pathways in *Enterobacter* sp. FY-07 when the carbon source was changed from glucose to glycerol. Red arrows indicate that the expression of genes encoding the enzymes related to this reaction was upregulated; green arrows indicate downregulation under the same conditions; A red square indicates the transcription level of the coding gene upregulated one fold; A green square indicates the transcription level of the coding gene downregulated one fold; An orange square indicates the transcription level of the coding gene upregulated 50 folds. Exact numerical results of relative fold changes were shown in Additional file 1: Figure S1. *UGPase* UDP-glucose pyrophosphorylase, *BCS* bacterial cellulose synthase, *HK* hexokinase, *GK* glucokinase, *PM* phosphoglucomutase, *PGI* phosphoglucoisomerase, *PFK* phosphofructokinase, *PGD* glycerophosphate dehydrogenase, *TPI* triose phosphate isomerase, *GADPH* glyceraldehyde-3-phosphate dehydrogenase, *SD* succinate dehydrogenase, *MD* malic dehydrogenase, *PGK* phosphoglycerate kinase, *PGM* phosphoglycerate mutase, *PK* pyruvate kinase, *PDC* pyruvate dehydrogenase complex

FY-07 produces BC when cultivated with various common sources of carbon, such as molasses, glucose (Fig. 1a), glycerol (Fig. 1b), and sucrose [21, 22]. The biosynthesis pathway of BC from glucose in FY-07 has been described in our previous study [21]; briefly, glucose is consecutively catalyzed by glucokinase (GK), phosphoglucomutase (PM), UDP-glucose pyrophosphorylase, and BC synthase complex, resulting in BC biosynthesis [21]. Inactivation of any enzyme involved in this pathway restricts the ability of FY-07 to produce BC. In addition, GK inactivation affects the normal metabolism of FY-07 as it is a key enzyme in the glycolysis pathway. A feasible strategy to construct a conditional BC-producing strain is thus to decrease the substrate (glucose) concentration in the BC biosynthesis pathway by reducing or inactivating the gluconeogenesis pathway in FY-07 [21]. To reduce the gluconeogenesis pathway, transcription levels of related genes involved in the glycolysis pathway, gluconeogenesis pathway, and glycerol metabolism were analyzed by qRT-PCR upon cultivating FY-07 with glucose or glycerol as the sole carbon source. In comparison to when glucose was used as the sole carbon source, when glycerol was used as the sole carbon source, the expression of genes encoding phosphofructokinase, phosphoglycerate kinase, phosphoglycerate mutase and pyruvate dehydrogenase complex was downregulated, however, the expression of genes encoding glycerol kinase, phosphoglycerol dehydrogenase, triose phosphate isomerase, glyceraldehyde-3-phosphate dehydrogenase, aldolase, fructose-1, 6-bisphosphatase (FBP), and phosphoglucose isomerase was upregulated (Fig. 2). The numerical results of relative fold change of these genes can be seen in Additional file 1: Figure S1.

According to the aforementioned results, the gene encoding FBP can be regarded as a target gene, for it catalyzing irreversible reactions in the gluconeogenesis pathway. However, completely inactivating this gene is undesirable because its catalytic product is also used to synthesize essential components of cells such as peptidoglycan and lipopolysaccharide [28]. Genomic sequence analysis suggested that the genome of FY-07 includes three genes (AKI40_1324, AKI40_4472, and AKI40_4858) encoding FBP isoenzymes. The results of qRT-PCR indicated that the transcriptional level of AKI40_4472 was higher than that of AKI40_4858 and AKI40_1324 when glycerol or glucose was the sole carbon source (Additional file 1: Figure S2), suggesting that FBP encoded by AKI40_4472 mainly contributes to the conversion of fructose-1, 6-bisphosphate to fructose-6-phosphate in FY-07. Therefore, AKI40_4472 was selected as the target gene to reduce the gluconeogenesis pathway of FY-07 for constructing a genetically engineered strain that could grow on glycerol and not synthesize substantial quantities of BC.

### Construction and characterization of the conditional BC-producing strain

A conditional BC-producing strain was constructed by knocking out AKI40_4472. PCR was used to confirm the successful knock out of this target gene (Additional file 1: Figure S3). We designated this strain as FY-0701. Its growth rate was slightly faster than that of FY-07 when glycerol was used as the sole carbon source (Additional file 1: Figure S4). SEM demonstrated that the BC-producing ability of FY-0701 remained unaffected when glucose was used as the sole carbon source (Fig. 1c); only a few BC fibers were observed when glycerol was the sole carbon source (Fig. 1d). These results suggested that FY-0701 could still synthesize sufficient glucose derivatives via the gluconeogenesis pathway, using FBP isoenzymes encoded by AKI40_4858 and AKI40_1324, to ensure cell growth rather than synthesizing large quantities of BC. Therefore, for microbial EOR (MEOR), preparation of injection cells of FY-0701 using glycerol as the sole carbon source can potentially save substantial costs as special equipment or cellulase is not required.

### Plugging capacity of FY-0701 in the core flooding experiment

Core flooding experiments have been extensively used to simulate and evaluate microbial selective plugging for MEOR practices [29–31]. Three types of cores are commonly used: sand-packed columns, artificial cores, and natural reservoir cores [15]. We chose artificial cores to evaluate the efficiency of selective plugging by FY-07 and FY-0701 because they are more economical than natural reservoir cores and more suitable for observation under a scanning electron microscope than sand-packed columns. To simulate the heterogeneity of reservoirs, we connected an artificial core with low permeability and another one with high permeability in parallel. In order to avoid the production of high volumes of biopolymers during the injection process, injection cells and nutrients are usually separately injected in the production practice in oil fields. Table 3 shows the details of the core flooding experiment.

**Fig. 3:**
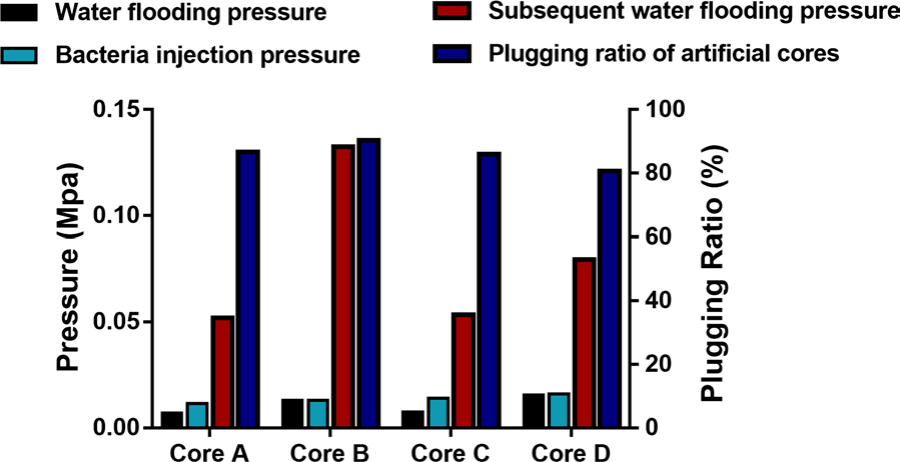
Comparison of key parameters in core experiments. Core A, B, C and D correspond to core A, B, C and D in Tables 3, 4. Core A with high permeability and Core C with low permeability were connected in parallel to evaluate the plugging effect of *Enterobacter* sp. FY-0701, while Core B with high permeability and Core D with low permeability were connected in the same way to evaluate the plugging effect of *Enterobacter* sp. FY-07. Water flooding pressure, bacteria injection pressure and subsequent water flooding pressure were recorded during the experiment process; The plugging ratios of artificial cores were calculated by the Eq. (1)

During the injection process, injection pressures of FY-07 were slightly higher than those of FY-0701, suggesting that residual BC fibers on FY-07 cells impeded their migration in artificial cores. After 3 days of incubation at 30 °C, although plugging ratios of both FY-07 and FY-0701 in all artificial cores were > 80%, subsequent water flooding pressures of artificial cores with high permeability had no obvious difference whereas the pressure of the core B plugged by FY-0701 was 66.67% higher than that of core D plugged by FY-07 (Fig. 3). These results suggested that the migration ability of FY-0701 in oil-bearing formations with low permeability was higher than that of FY-07. The reduction in the permeability of artificial cores represented by the increase in the plugging ratio demonstrated that both FY-07 and FY-0701 could be used to redirect the flooding water into low permeability zones where the oil was left.

### BC production and location of the plugging position of FY-0701 in artificial cores

Microbial plugging has great potential in EOR, but the plugging position may largely influence the sweep volume of flooding water, consequently affecting oil recovery [18, 32]. The results of a mathematical model showed that high permeability zones are plugged when biopolymers are produced in situ in oil reservoirs [33]. Flooding water is then redirected into low permeability zones, thereby enlarging sweeping volume [16, 34]. The deeper the plugging position, the larger is the volume between the plugging position and entrance, and the higher is the sweep volume [25, 26]. This implies that the sweep volume of flooding water in deep profile control was higher in comparison to non-deep profile control. Accordingly, oil displacement efficiency should be improved in deep profile control than in non-deep profile control [35].

**Fig. 4:**
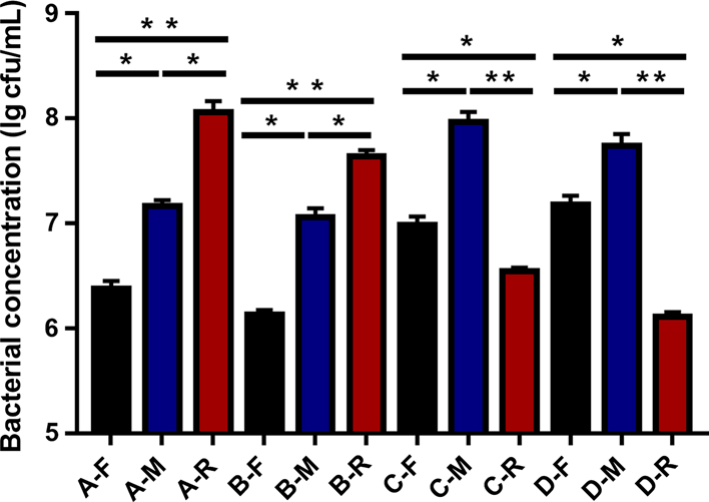
Bacterial concentration in the front-, middle-, and rear-ends of artificial cores A, B, C and D. Core A, B, C and D correspond to core A, B, C and D in Fig. 3 and Tables 3, 4. Black, blue, and red columns represent the bacterial concentration in the front-, middle-, and rear-ends, respectively. The results represent means from three replicates in the same experiments. *p < 0.05; **p < 0.01. A-F, the front-end of core A; A-M, the middle-end of core A; A-R, the rear-end of core A; B-F, the front-end of core B; B-M, the middle-end of core B; B-R, the rear-end of core B; C-F, the front-end of core C; C-M, the middle-end of core C; C-R, the rear-end of core C; D-F, the front-end of core D; D-M, the middle-end of core D; D-R, the rear-end of core D

In practice, it is difficult to measure variations in sweep volume and profile control both in oil reservoirs and artificial cores. Therefore, cell concentration and BC production in the front-, middle-, and rear-ends of artificial cores were quantified to determine the deep profile control ability of FY-0701 and FY-07. The obtained results indicated that the cell concentration of FY-0701 gradually increased from the front- to rear-ends of the cores, while the highest cell concentration of FY-07 was observed at the middle-end of the core (Fig. 4).

**Fig. 5:**
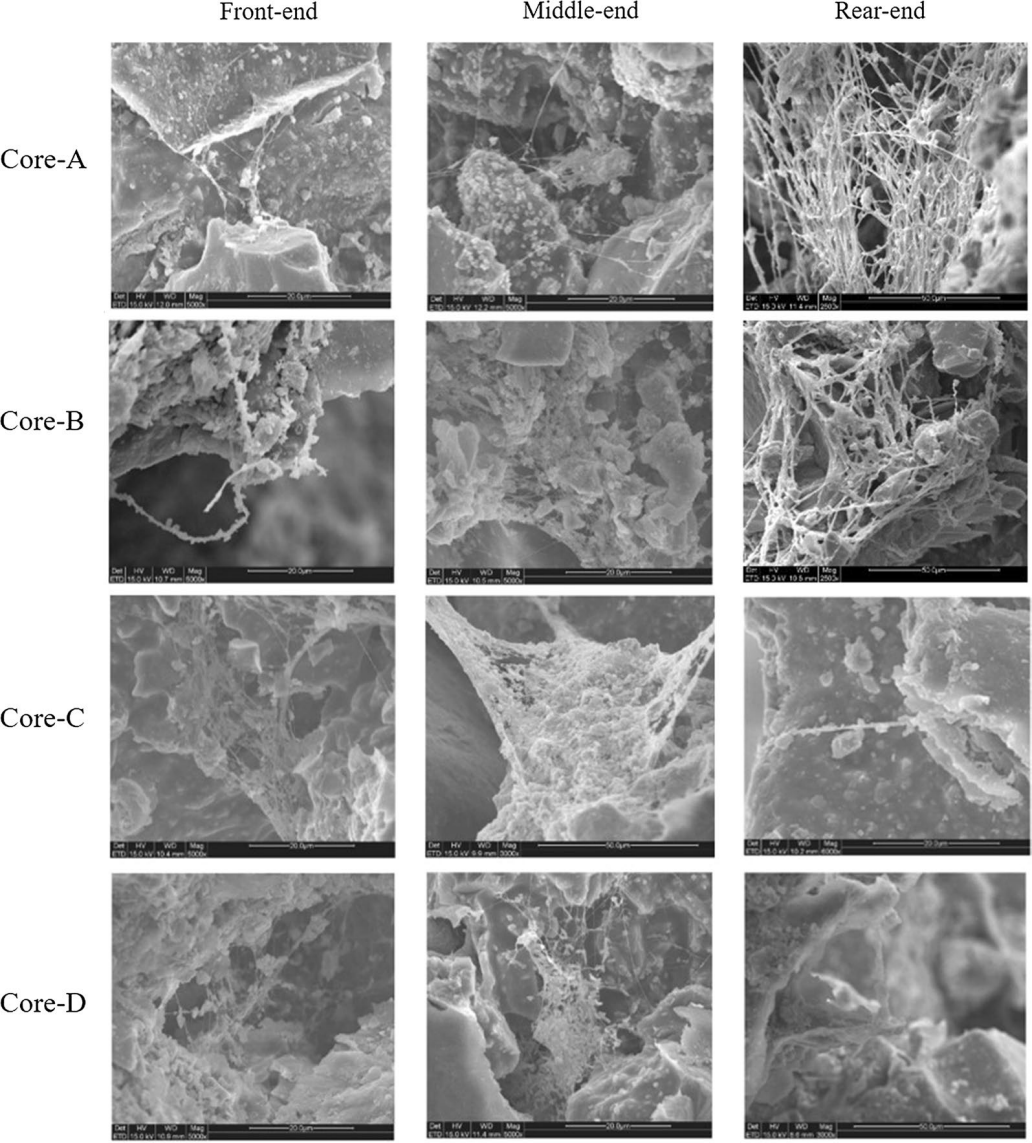
Comparison of BC production in the front-, middle-, and rear-ends of artificial cores by SEM observation. Core A, B, C and D correspond to core A, B, C and D in Figs. 3, 4 and Tables 3, 4. The magnification is 5000×

To visually compare the deep profile control abilities of FY-07 and FY-0701, BC production in artificial cores was observed by SEM (Fig. 5). In accordance with variations in cell concentration, the quantity of BC in artificial cores plugged by FY-0701 (Fig. 5, cores A and B) also showed a gradual increment. These results indicated that FY-0701 is more suitable for deep profile control than FY-07.

### EOR of the genetically engineered strain in oil displacement experiment

**Fig. 6:**
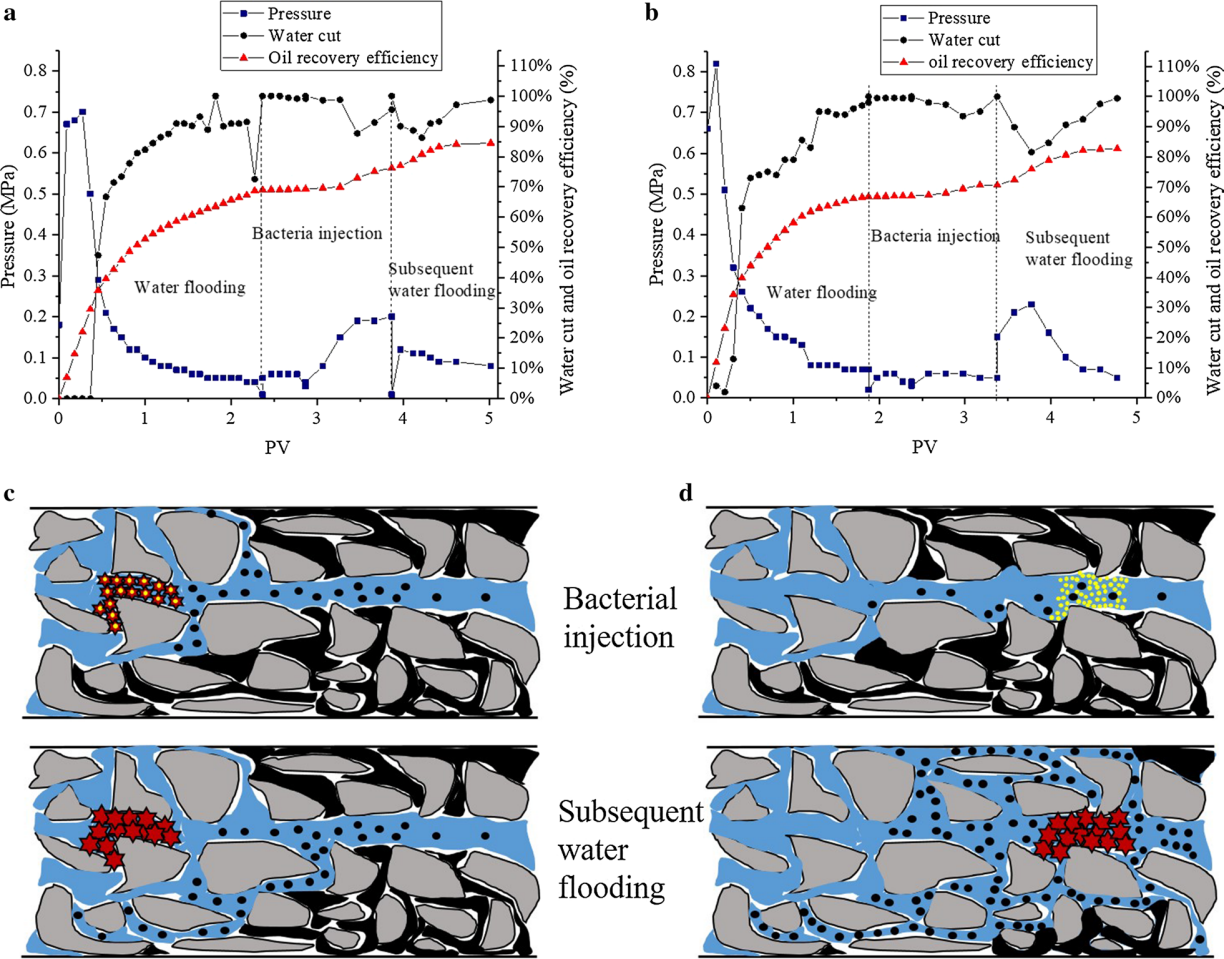
Key parameters and the schematic diagram of oil displacement experiments using *Enterobacter* sp. FY-07 and FY-0701. **a** Pressure, water cut, and oil recovery efficiency of column A; **b** Pressure, water cut, and oil recovery efficiency of column B; **c** The schematic diagram of *Enterobacter* sp. FY-07 in oil displacement process. **d** The schematic diagram of *Enterobacter* sp. FY-0701 in oil displacement process. Column A and B correspond to column A and B in Table 4. Pressure was recorder during the experiment process; Water cut was calculated by the Eq. (3); The oil recovery efficiency was calculated by the Eqs. (2) and (4). Gray polygons indicate porous medium, blue area indicates water flow, black area indicates crude oil, yellow circles indicate bacteria, red stars indicate BC, and black circles indicate crude oil that was displaced

The ultimate goal of deep profile control is to increase the sweep volume of injected water and EOR in low permeability zones. Therefore, the oil displacement abilities of FY-07 and FY-0701 were compared in the core flooding experiment using two sand-packed columns with similar permeability and PV. During the subsequent water flooding process, EOR increased to 12.09% in the sand-packed column plugged by FY-0701, while that of FY-07 plugged sand-packed column only increased to 8.23% (Fig. 6). In addition, during the cell injection process, the injection pressure of FY-07 rapidly increased to its highest, becoming approximately 3.16-fold that of FY-0701. In comparison with the water flooding process, the injection pressure of subsequent water flooding only increased by twofold. These results indicated that the residual BC fibers on FY-07 cells can considerably increase the difficulty and cost of MEOR techniques, as they can contribute toward the retention of bacterial cells at the region near injection wells and increase injection pressure. Unlike FY-07, pressure when using FY-0701 did not show an obvious change during the cell injection process, but it increased by approximately 3.29-fold in the subsequent water flooding process (Fig. 6a, b), which conforms to the general trait of profile control [10, 16, 26]. The schematic diagram of FY-07 and FY-0701 in oil displacement processes are shown in Fig. 6c, d.

Although premature plugging in the core flooding experiment could increase ORE during the injection process (Fig. 6a), this is difficult to achieve in a long-term water-flooded reservoir due to less quantity of residual oil near the injection wells [7]. Moreover, with the increase of injection time, the drastic increase of injection pressure will be more serious, which will affect the subsequent injection. In addition, biopolymer production during the injection process can inevitably cause plugging in supply pipes and walls of the well bore [16, 27]. Deep profile control may thus be more effective for long-term water flooding reservoirs. Our results indicated that during the subsequent water flooding process, EOR increased to 12.09% in the sand-packed column plugged by FY-0701, which was 3.86% higher than when the column was plugged by FY-07 (Fig. 6). This implies that FY-0701 is more suitable for deep profile control than FY-07.

Considering the size of sand-packed columns, it is challenging to completely simulate the injection process in an oil field. Bacterial cells could sufficiently be injected in our core flooding experiment, but in actual production practice, proliferation of bacterial cells is essential during the injection process to reduce the mining cost. FY-0701 is therefore a good candidate for deep profile control in oil reservoirs. Glycerol-based nutrient solutions can supply nutrients to ensure FY-0701 growth and transport the cells to high permeability zones in oil-bearing formations. Also, glucose-based nutrient solutions can activate in situ BC-producing ability of FY-0701 to increase sweep volume and enhance oil recovery by reducing the permeability of such formations.

## Conclusions

In this study, a genetically engineered strain FY-0701 which could control producing BC or not by using different carbon source was constructed. Core flooding experiments for profile control indicated that this conditional BC-producing strain could reach deep zones of reservoirs to reducing their permeability, and EOR increased to 12.09% during the subsequent water flooding process in oil displacement experiments. It suggested that FY-0701 is promising for deep profile control in MEOR techniques. This study has important guiding significance for the subsequent design and application of genetically engineered strains in industrial application.

## Materials and methods

### Bacterial strains, plasmids, and culture conditions

Bacterial strains and plasmids used in this study are listed in Table 1. *Escherichia coli* S17 was used for plasmid construction and conjugation; it was grown on LB medium (per liter, 10 g peptone, 5 g yeast extract, and 5 g sodium chloride) at 37 °C. FY-07 (CGMCC No. 6103), isolated from the production water of the Jilin oilfield (China Petroleum Natural Gas Co., Ltd., Jilin Oil Field Branch), was used for genetic engineering. FY-07 is a BC-producing, carbenicillin-resistant, tetracycline-sensitive, Gram-negative, facultative anaerobic, rod-shaped bacterium. Cell suspension of FY-07 or FY-0701 was prepared as previously described [22].

**Table 1:** Strains and plasmids used in this study

Strains and plasmids	Description	References
*Escherichia coli* S17	recA pro hsdR RP4-2-Tc::Mu-Km::Tn7	[40]
*Enterobacter* sp. FY-07	Wild-type strain, BC-producing strain	[22]
*Enterobacter* sp. FY-0701	AKI40_4472 deletion derivative of *Enterobacter* sp. FY-07	This work
pTSK1	Ts; sacB; Ampr; Tcr; oriT	[21]
pMD19T-*bcsA*	T vector containing *bcsA* gene	This work

To prepare *Enterobacter* sp. cells for core-flooding, cell suspension was inoculated into 100 mL fermentation medium (per liter, 2 g KNO_3_, 2 g NH_4_Cl, 1 g KH_2_PO_4_, 0.5 g K_2_HPO_4_·3H_2_O, and 0.25 g MgSO_4_·7H_2_O) containing 30 g/L glycerol as the sole carbon source and cultured with shaking at 30 °C until the late logarithmic growth phase. Subsequently, bacterial cells were collected by centrifugation at 6000×*g* for 10 min and diluted using distilled water for core flooding and oil displacement experiments. In addition, 1 g/L cellulase was added to the fermentation medium when FY-07 was cultivated for RNA extraction, gene knockout experiments, and preparation of cells for injection. A temperature-sensitive (Ts) plasmid, pTSK1, was used as the vector for gene knockout. The culture was grown at 42 °C for Ts plasmid curing. Ampicillin (100 μg/mL), tetracycline (15 μg/mL), or carbenicillin (50 μg/mL) was added to the medium for screening of transformants.

### DNA/RNA extraction and quantitative real-time reverse transcription PCR (qRT-PCR)

DNA/RNA extraction and qRT-PCR were performed as previously described [21, 36]. RNA extraction and qRT-PCR were used to analyze the transcriptional levels of key genes involved in central carbon metabolic pathways when glucose or glycerol was used as the sole carbon source. Briefly, cDNA of bacteria cultured using glucose or glycerol as the sole carbon source was synthesised using a Quantscript RT Kit (Tiangen, Beijing, China), following the manufacturer’s instructions. A total input of 500 ng of RNA and random hexamers were used in each reaction. The 16S rRNA gene was used as the endogenous reference gene. The ratio of expression was quantified by 2^−ΔΔCT^ method. Three replicates were analyzed for each gene. The error of the R-values (ΔR) was calculated by the Bio-rad qPCR software. To calculate the fold of expression levels of key genes when glycerin was used as the carbon source, the expression levels of these genes of the sample using glucose as the carbon source were used as the control. DNA extraction and qRT-PCR were performed to evaluate the deep profile control ability of FY-07 or FY-0701 by measuring the concentration of bacteria in the front-, middle-, and rear-ends of artificial cores. pMD19T-*bcs*A was used for the preparation of standard curve in absolute quantification. Primers 895-FW/895-Rv were used to quantify the amount of *bcs*A gene in the samples representing the concentration of bacteria. All primers used for qRT-PCR are presented in Additional file 1: Table S1.

### Construction and evaluation of the genetically engineered strain

Gene knockout was achieved by homologous recombination. To construct the gene knockout vector, 1.5 kb upstream and downstream flanking sequences of the target gene were amplified from the chromosome of *Enterobacter* sp. FY-07. The DNA fragments were connected by overlap PCR [37]. The resulting fragment was then digested with restriction enzymes and ligated into pTSK1 treated with the same enzymes. The resulting gene knockout vector was introduced into FY-07 via conjugation [38], and transformants were selected and verified as previously described [21]. The representative diagram of gene-knockout was shown in Additional file 1: Fig. S5. Non-toxic dye Super GelRed added in agarose gel was purchased from US Everbright Inc. (Suzhou, China). The genetically engineered strain was named FY-0701. All primers used in its construction and determination are listed in Additional file 1: Table S2. FY-0701 was evaluated by determining its growth using glycerol as the sole carbon source, and its ability to produce BC was assessed using SEM.

### Core flooding experiment

Detailed information pertaining to artificial cores used in this study is listed in Table 2. The cores were inserted into metal core holders and saturated with oil-field injection water of Luliang after vacuum pumping. Pore volume (PV) was calculated using the volume of saturating water. The saturated cores were then flooded with injection water until the pressure became stable to measure permeability. A low permeability core and a high one were connected in parallel to simulate the heterogeneity of reservoirs, and 0.5 PV of media inoculated with bacterial cells was then injected. Subsequently, 1 PV of fermentation medium containing 30 g/L glucose was injected into the cores; after the injections, distilled water was used to promote the bacterial cell suspension and fermentation medium to achieve deep profile control (Table 3). After incubation for 3 days at 30 °C, the cores were flooded with the same injection water until the pressure stabilized. The flow rate for the flooding was set at 1 mL/min. Pressure was recorded to determine effective permeability before (*P*_*before*_) and after (*P*_*after*_) bacterial treatment, and the blocking ratio (*R*) was calculated using the following equation:

1 $$R = \left( {{\rm{1}} - {P_{after}}/{P_{before}}} \right) \times 100\%$$

**Table 2:** Parameters of artificial cores used in core flooding experiment

Core number	Inner diameter (cm)	Length (cm)	Pore volume (PV, mL)	Porosity (Φ, %)	Permeability to water (Kw, mD)
Core-A	3.8	29.5	72.2	21.6	619
Core-B	3.8	29.2	70.1	21.2	330
Core-C	3.8	29.4	73.4	22.0	576
Core-D	3.8	28.8	70.3	21.5	273

**Table 3:** Injection formula used in core flooding experiment

Connection type	Injection formula	Strain
Core-A and Core-B (in parallel)	0.5PV BS + 1PV FM + 0.5PV DW	Genetically engineered strain
Core-C and Core-D (in parallel)	0.5PV BS + 1PV FM + 0.5PV DW	Wild-type strain

*BS* bacterial seed solution was diluted using distilled water to a concentration of 4%, *FM* fermentation medium with glucose as the carbon source, *DW* distilled water

### Scanning electron microscope observation

Scanning electron microscope was used to determine the ability of FY-07 and FY-0701 to produce BC when cultivated using glucose or glycerol as the sole carbon source. Fermentation medium plates were cut into 3 mm × 3 mm for SEM observation. To analyze the selective plugging position, the front-, middle-, and rear-ends of artificial cores were cut at 5, 15, and 25 cm away from the injection site and sampled separately. Samples were then analyzed using SEM. After freeze-drying, samples were mounted on aluminum studs and coated with a gold/palladium alloy under high vacuum conditions [39]. QUANTA200 scanning electron microscope (FEI, Oregon, USA) was used for microscopic observation.

### Oil displacement experiment

Oil displacement efficiency of both FY-07 and FY-0701 was tested using sand-packed columns. To simulate reservoirs as much as possible, long sand-packed columns (inner diameter 38 mm, length 500 mm) were selected for oil displacement experiment. 50 mesh and 325 mesh quartz sands were mixed in a ratio of 3 to 1 and filled into two stainless steel columns to obtain two columns with similar permeability (column A, 1369 cm/day; column B, 1285 cm/day) and PV (column A, 220 mL; column B, 200 mL; Table 4). Both columns were saturated with injection water from Lu-9 block after vacuum pumping. Crude oil from Luliang oil field was injected into each column until it reached irreducible water saturation. The physical and chemical properties of the crude oil obtained from the Luliang Oilfield were shown in Additional file 1: Table S3. The columns were then flooded again with injection water until > 98% water cut in effluent was obtained. Subsequently, 0.5 PV of media inoculated with FY-07 or FY-0701 (10^9^ cells/mL) cultivated using glycerol as the carbon source was injected into the columns; furthermore, 1 PV of fermentation medium containing 30 g/L glucose was injected into both columns. After incubation for 3 days at 30 °C, the same injection water was used for the subsequent water flooding process. The flow rate for the flooding was set at 1 mL/min. The volume of released oil, water cut, and pressure in the effluent were measured. Oil recovery efficiency (ORE, %) was calculated as follows:

**Table 4:** Parameters of sand-packed columns used in oil displacement experiment

Parameter	Inner diameter (cm)	Length (cm)	Pore volume (PV, mL)	Irreducible water saturation (Swc, %)	Porosity (Φ, %)	Permeability to gas (Kg, mD)
Column A	3.8	50	220	24.32	38.82	1369
Column B	3.8	50	200	15.25	35.29	1285

2 $${\text{ORE}} \; (\% ) = \frac{{{\text{Total volume of oil recovery}}}}{{{\text{Original oil in place}}}} \times 100$$

wherein “Original oil in place” (mL) is the volume of water displaced by oil saturation.

Water cut (%) was derived as follows:

3 $${\text{Water cut}} \; (\% ) = \frac{{{\text{Volume of water}}}}{{{\text{Volume of production liquid}}}} \times 100$$

EOR was estimated using the following formula:

4 $${\text{EOR}}\;(\% ) = {\text{ORE}}_\text{m} - {\text{ORE}}_\text{w}$$

wherein ORE_m_ is the ORE at the end of subsequent water flooding and ORE_w_ is the ORE at the end of bacterial injection [10].

### Statistical analysis

All experiments were performed in triplicate if conditions permit. The significance of the data was evaluated using the generalized linear model (GLM) with p < 0.05 indicating significance (Minitab ANOVA Statistical software, Release 13.30, Penn State University-Park, PA).

## Supplementary information

**Additional file 1: Figure S1.** The relative fold change of genes involved in central carbon metabolic pathways under the conditions using glycerol as the sole carbon source. The expression of these genes of the sample using glucose as the carbon source were used as a control. *p < 0.05. The results represent means from three replicates in the same experiments. **Figure S2.** The expression level of three genes (AKI40_1324, AKI40_4858 and AKI40_4472) encoding for FBP isoenzymes under the conditions using glucose or glycerol as the sole carbon source. The expression level of AKI40_4858-Glucose was used as control. *p < 0.05. The results represent means from three replicates in the same experiments. **Figure S3.** Gene deletion confirmation. Genomic DNA from strains *Enterobacter* sp. FY-0701 and FY-07 was probed for the presence of the deletion target genes by PCR. Lane 1, DL2000 DNA Marker (Takara); Lane 2, *Enterobacter* sp. FY-0701; Lane 3, *Enterobacter* sp. FY-07. **Figure S4.** Growth curves of *Enterobacter* sp. FY-07 and FY-0701 under the condition using glycerol as the sole carbon source. The results represent means from three replicates in the same experiments. **Figure S5.** The representative diagram of gene-knockout in *Enterobacter* sp. FY-07. *tet*A, tetracycline efflux protein encoding gene; *sac*B, levansucrase encoding gene; *ori*T, incP origin of transfer; *rep*A101ts, the gene encoding temperature-sensitive protein needed for replication with the *ori*R101; *ori*R101, low-copy replication origin that requires the Rep101 protein; Tc, tetracycline; Amp, Ampicillin; LB, Luria-Bertani medium; Suc, sucrose. **Table S1.** Primers used in qRT-PCR. **Table S2.** Primers used in construction and determination of genetically engineered strain. **Table S3.** The physical and chemical properties of the crude oil obtained from the Luliang Oilfield.

## Data Availability

The datasets supporting the conclusions of this article are included within the article.
