# Supplementary material for: Microbial enhanced oil recovery through deep profile control using a conditional bacterial cellulose-producing strain derived from Enterobacter sp. FY-07
Source: Microb Cell Fact. 2020 Mar 5;19:59. doi: 10.1186/s12934-020-01314-3 (PMC7059367; doi:10.1186/s12934-020-01314-3)
Supplement: Supplementary file 1 — Additional file 1: Figure S1. The relative fold change of genes involved in central carbon metabolic pathways under the conditions using glycerol as the sole carbon source. The expression of these genes of the sample using glucose as the carbon source were used as a control. *p < 0.05. The results represent means from three replicates in the same experiments. Figure S2. The expression level of three genes (AKI40_1324, AKI40_4858 and AKI40_4472) encoding for FBP isoenzymes under the conditions using glucose or glycerol as the sole carbon source. The expression level of AKI40_4858-Glucose was used as control. *p < 0.05. The results represent means from three replicates in the same experiments. Figure S3. Gene deletion confirmation. Genomic DNA from strains Enterobacter sp. FY-0701 and FY-07 was probed for the presence of the deletion target genes by PCR. Lane 1, DL2000 DNA Marker (Takara); Lane 2, Enterobacter sp. FY-0701; Lane 3, Enterobacter sp. FY-07. Figure S4. Growth curves of Enterobacter sp. FY-07 and FY-0701 under the condition using glycerol as the sole carbon source. The results represent means from three replicates in the same experiments. Figure S5. The representative diagram of gene-knockout in Enterobacter sp. FY-07. tetA, tetracycline efflux protein encoding gene; sacB, levansucrase encoding gene; oriT, incP origin of transfer; repA101ts, the gene encoding temperature-sensitive protein needed for replication with the oriR101; oriR101, low-copy replication origin that requires the Rep101 protein; Tc, tetracycline; Amp, Ampicillin; LB, Luria-Bertani medium; Suc, sucrose. Table S1. Primers used in qRT-PCR. Table S2. Primers used in construction and determination of genetically engineered strain. Table S3. The physical and chemical properties of the crude oil obtained from the Luliang Oilfield. [file 12934_2020_1314_MOESM1_ESM.docx]

**Microbial enhanced oil recovery through deep profile control using a conditional bacterial cellulose-producing strain derived from *Enterobacter* sp. FY-07**

Ge Gao^a, 1^, Kaihua Ji ^b, 1^, Yibo Zhang ^a^, Xiaoli Liu ^c^, Xuecheng Dai ^c^, Bo Zhi ^a^, Yiyan Cao ^a^, Dan Liu ^a^, Mengmeng Wu ^a^, Guoqiang Li ^a,^ #, Ting Ma ^a,^ #

^a^ *Key Laboratory of Molecular Microbiology and Technology, Ministry of Education, College of Life Sciences, Nankai University, Tianjin, 300071, PR China*

^b^ *Tianjin Key Laboratory of Radiation Medicine and Molecular Nuclear Medicine, Department of Radiobiology, Institute of Radiation Medicine of Chinese Academy of Medical Science & Pecking Union Medical College, Tianjin, 300192, PR China*

^c^ *Research Institute of Experiment and Detection, Xinjiang Oilfield Branch Company, PetroChina, Karamay, Xinjiang, 834000, PR China*

#Corresponding author.

E-mail address: tingma@nankai.edu.cn (T. Ma), gqli@nankai.edu.cn (G.Q. Li).

**
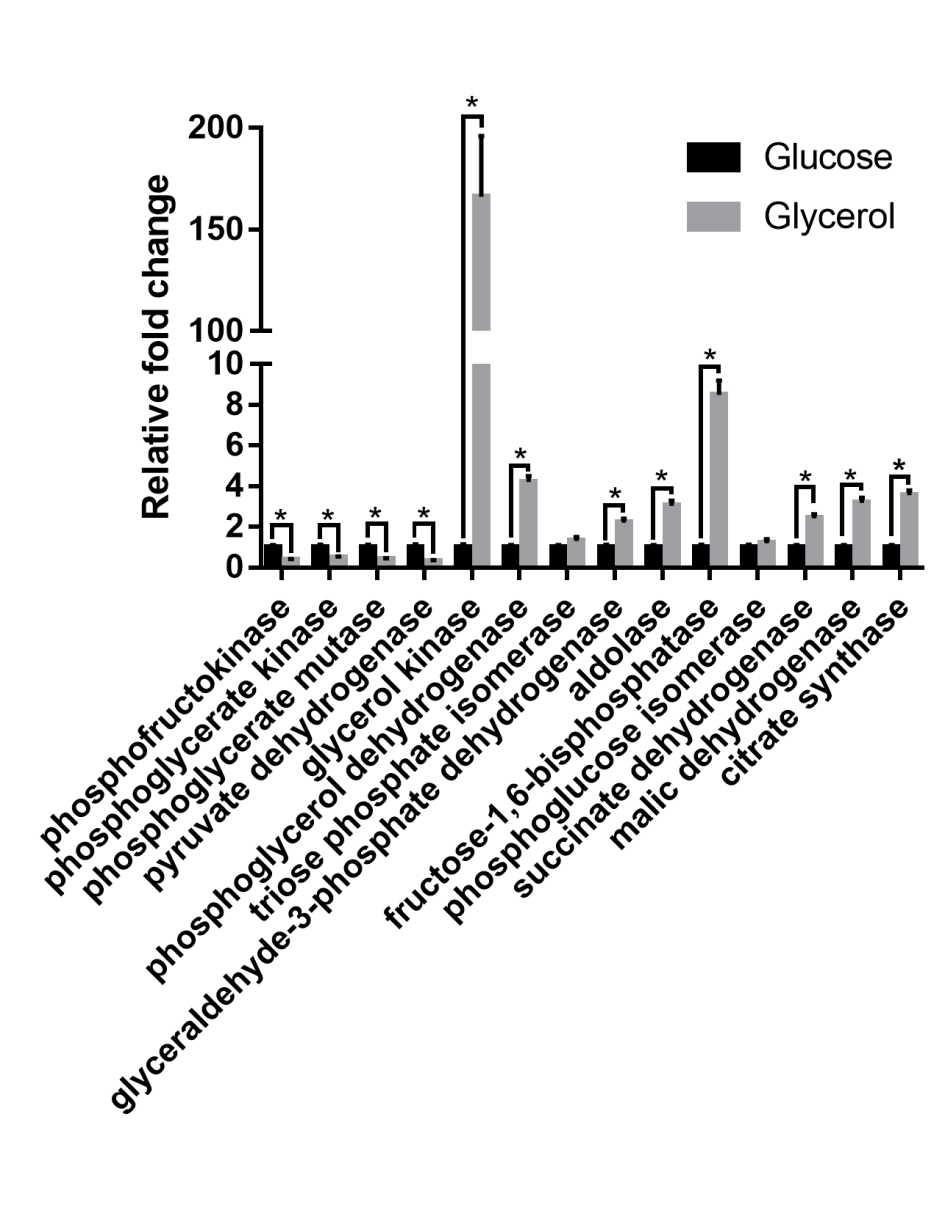
**

**Figure S1** The relative fold change of genes involved in central carbon metabolic pathways under the conditions using glycerol as the sole carbon source. The expression of these genes of the sample using glucose as the carbon source were used as a control. * *p*<0.05. The results represent means from three replicates in the same experiments.


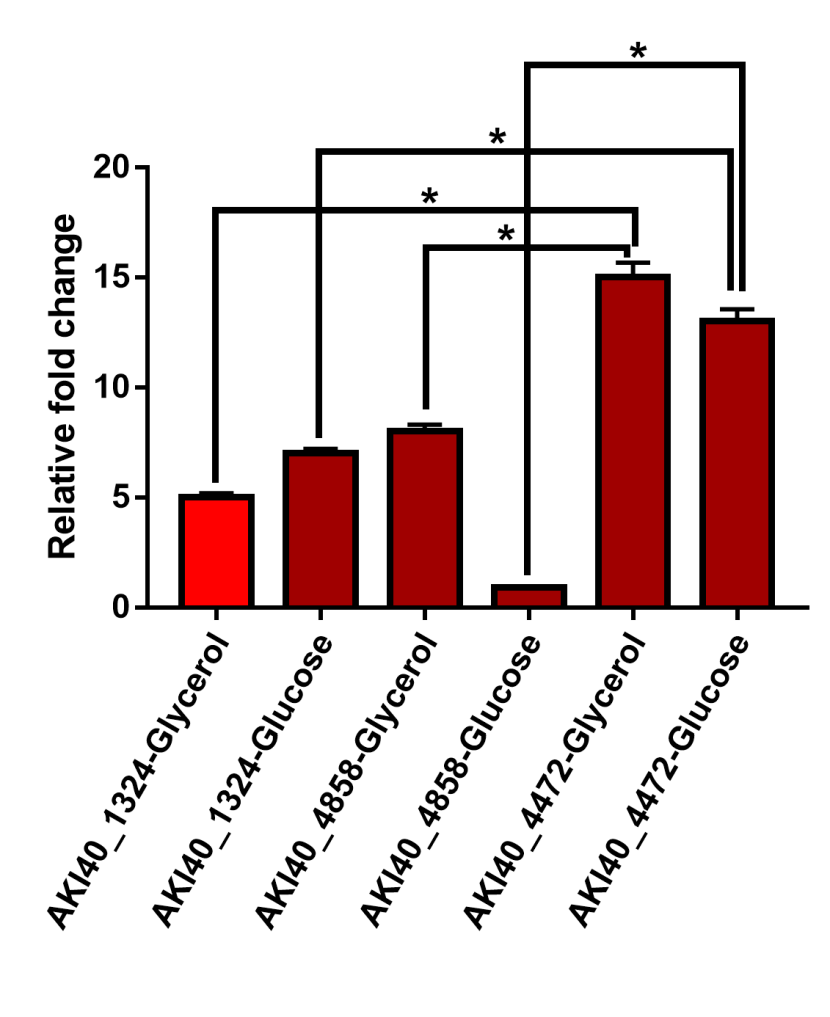
**Figure S2** The expression level of three genes (AKI40_1324, AKI40_4858 and AKI40_4472) encoding for FBP isoenzymes under the conditions using glucose or glycerol as the sole carbon source. The expression level of AKI40_4858-Glucose was used as control. * *p*<0.05. The results represent means from three replicates in the same experiments.

**
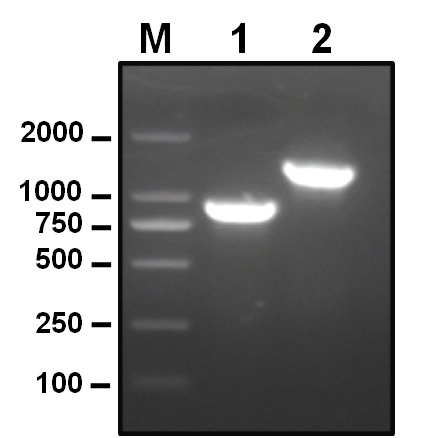
 Figure S3** Gene deletion confirmation. Genomic DNA from strains *Enterobacter* sp. FY-0701 and FY-07 was probed for the presence of the deletion target genes by PCR. Lane 1, DL2000 DNA Marker (Takara); Lane 2, *Enterobacter* sp. FY-0701; Lane 3, *Enterobacter* sp. FY-07.


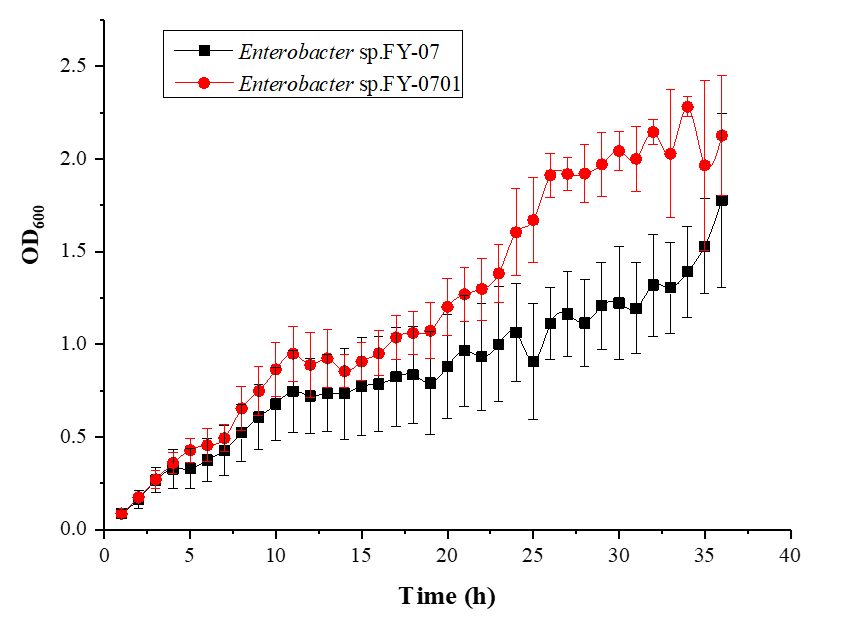


**Figure S4** Growth curves of *Enterobacter* sp. FY-07 and FY-0701 under the condition using glycerol as the sole carbon source. The results represent means from three replicates in the same experiments.


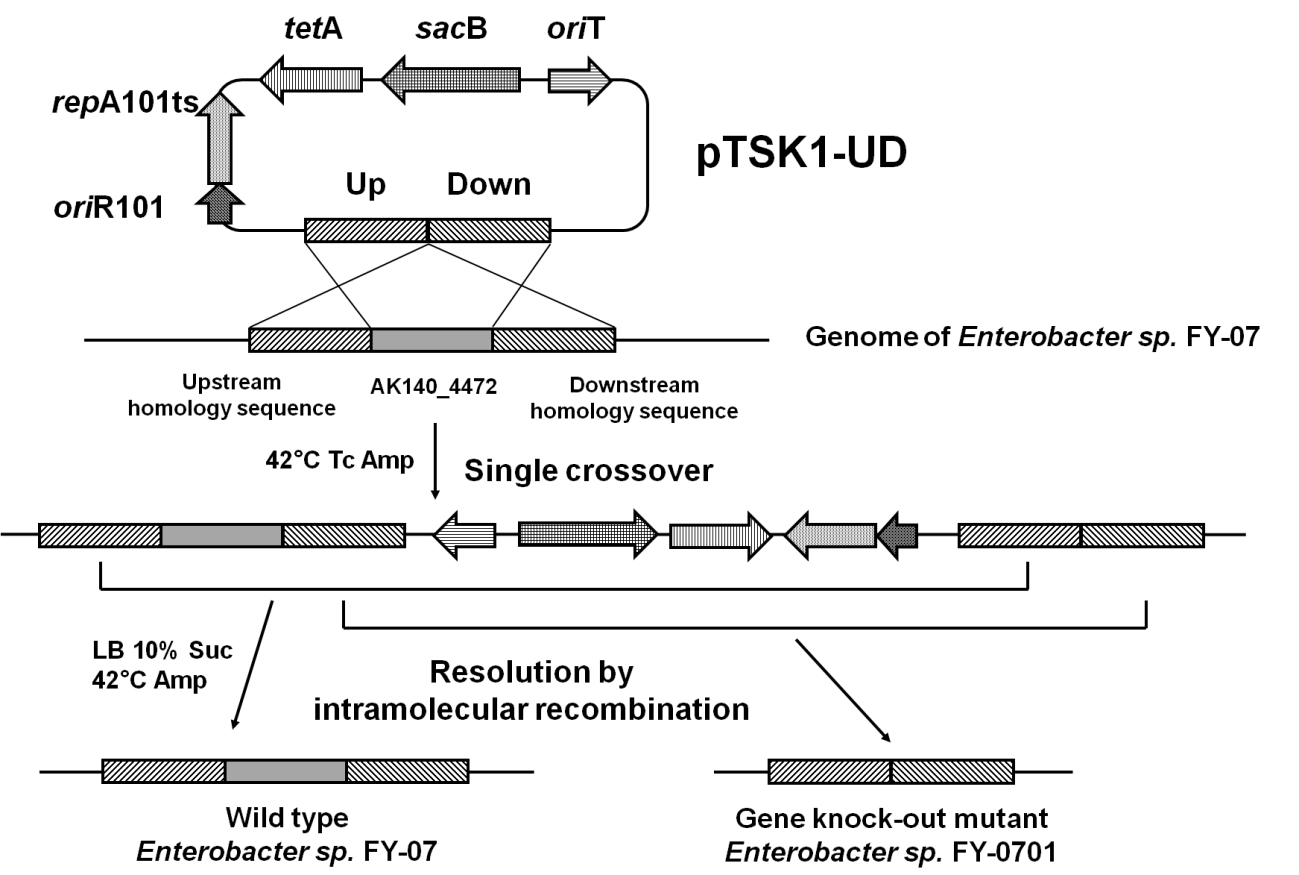


**Figure S5** The representative diagram of gene-knockout in *Enterobacter* sp. FY-07. *tet*A, tetracycline efflux protein encoding gene; *sac*B, levansucrase encoding gene; *ori*T, incP origin of transfer; *rep*A101ts, the gene encoding temperature-sensitive protein needed for replication with the *ori*R101; *ori*R101, low-copy replication origin that requires the Rep101 protein; Tc, tetracycline; Amp, Ampicillin; LB, Luria-Bertani medium; Suc, sucrose.

**Table S1** Primers used in qRT-PCR

| **Primers** | **Sequence (5’-3’)** | **Quantification of gene encoding for** |
| --- | --- | --- |
| 137-Fw | TTTGTCGCCAACGTAGATCA | Phosphoglycerate mutase |
| 137-Rv | CATCACCAGCGGCAAATAC |  |
| 399-Fw | GGATGCGTTCGGTGAGGT | Phosphoenolpyruvate carboxykinase |
| 399-Rv | GGTGGTCTGATAAAGGCAAAGGT |  |
| 827-Fw | CTGAAGCAGGGTGCGAAAGT | Phosphoglycerate kinase |
| 827-Rv | GCAGCGTATTTTTTGGAGAGTG |  |
| 828-Fw | CCAGGTAGCGAAAGAAAACAA | Fructose-bisphophate aldolase, class II |
| 828-Rv | CACCGTAATGTTCAGCCATC |  |
| 1324-Fw | CGGGTTACCTGCGACGATG | Fructose-1,6-bisphosphatase |
| 1324-Rv | GCCAAACAGCACGCCACTA |  |
| 1531-Fw | CGGTAGGCGAACTGTATGCG | Putative Glyceraldehyde-3-phosphate dehydrogenase-like |
| 1531-Rv | ATCACCAGCGGCACATCC |  |
| 1706-Fw | GCCTGCGGTGTTGCGTAATAT | Fructose-bisphosphate aldolase class 1 |
| 1706-Rv | GGCGTACAGCGTCTGGTCAT |  |
| 2201-Fw | CGGTTTGAAGTTTTCGTCGA | Phosphoglycerate mutase 1 family |
| 2201-Rv | AAGATGATGAGCGTTTCCCG |  |
| 3256-Fw | CTCAACGGCAAACTGAAAGG | Glyceraldehyde-3-phosphate dehydrogenase, type I |
| 3256-Rv | CGTCTGGGTGGCATCAAAT |  |
| 3571-Fw | GGCTTCGCAGAACCAAAATC | Pyruvate kinase II |
| 3571-Rv | CCTTCGCCTTTACCCAGGT |  |
| 4050-Fw | GCAAAGCCAGTCCAGTCAG | Enolase |
| 4050-Rv | GTTAAAGCAGCAGGTTACGAGC |  |
| 4275-Fw | CGTCTCCTGCGGTTTCCAC | Probable phosphoglycerate mutase *gpm*B |
| 4275-Rv | ACAGCGGATGGTCGTATTCC |  |
| 4472-Fw | CGGGTGAACTGACTGCTTTG | Fructose-1,6-bisphosphatase class 1 |
| 4472-Rv | CGATGTTGGAAGAGCCATCC |  |
| 4682-Fw | GTTGCCCTGCTGGAAGTAA | Glucose-6-phosphate isomerase |
| 4682-Rv | GGATTGGGTAGGTGGTCGTTA |  |
| 4858-Fw | CCGCTATGGGTCAGGCAAAT | Fructose-1,6-bisphosphatase, class II |
| 4858-Rv | TGGCATCGTGGCGAGGTT |  |
| 4862-Fw | GTCACTCTGAGCGTCGTACTTACC | Triosephosphate isomerase |
| 4862-Rv | GTGCCGATAGCCCAAACC |  |
| 551-Fw | AGTGGTTGAAGCGAAAGCGG | Malate dehydrogenase |
| 551-Rv | TGTAGCGTCTCCAGCATGCC |  |
| 2176-Fw | CAGCGATACAAGCGAACGG | Citrate synthase |
| 2176-Rv | CGCAATGACCTCTCCTACGC |  |
| 2179-Fw | TGCTTGACCTGGTGGTATTTG | Succinate dehydrogenase, flavoprotein subnit |
| 2179-Rv | GGAAGACCGAGAAGTTGTGCT |  |
| 2180-Fw | TGAAGAAGGGCGTGACATGA | Succinate dehydrogenase iron-sulfur protein |
| 2180-Rv | TTCTCATATTGGGCATAGAATTGC |  |
| 2181-Fw | CATACCGCTTCCGTGGTCAC | Oxoglutarate dehydrogenase, E1 component |
| 2181-Rv | TCCGTGCTGGTGATGTGCATAT |  |
| 2182-Fw | TGTTCCCGACCTGCCTGAGT | Dihydrolipoyllysine-residue succinyltransferase, E2 |
| 2182-Rv | TGCCAGAGCTGTTGCCTTCA |  |
| 2183-Fw | TAAAATGCCAGGTCCATGCG | Succinyl-CoA ligase subunit beta |
| 2183-Rv | GATTTCCACGCCACCTTCTGT |  |
| 2184-Fw | CTTATGGTACGCAGATGGTTGG | Succinyl-CoA ligase subunit alpha |
| 2184-Rv | TCACTTTCACCGTCAGCATATC |  |
| 2659-Fw | CCGCTGTGGAAAAAGCCTA | Isocitrate dehydrogenase |
| 2659-Rv | TATCGGTCAGTTCAGGGTGTT |  |
| 3010-Fw | GGCGAACCGTAGACCGA | Malate dehydrogenase |
| 3010-Rv | CCCTGCGACATCATTACCTG |  |
| 3315-Fw | GCATTGCGGCGTCATAAATC | Aconitate hydratase 1 |
| 3315-Rv | CCCTTTTTTGATGATATGGGGG |  |
| 3457-Fw | GTTCAGCACTTCGGTCTCG | Fumarate reductase, flavoprotein subunit |
| 3457-Rv | GCGGATAAACTGGGTATGGAC |  |
| 3459-Fw | ATCTGCTGAGCCGTGAATATGTTT | Tartrate/fumarate subfamily Fe-S type hydrolase beta subunit |
| 3459-Rv | TATCCAGCGGGGCGTTCT |  |
| 4162-Fw | GCTCCGCAGATGCCAGGTAA | Aconitate hydratase 2 |
| 4162-Rv | GGACATCAAAGAGCCGATCCTCT |  |
| 4165-Fw | ACCGATGCTGGAGATGG | Dihydrolipoyllysine-residue acetyltransferase component of pyruvate dehydrogenase C |
| 4165-Rv | ATCAACATTGGTGTGGCG |  |
| 4545-Fw | GCTATCGCTGGTCGTGCC | Fumarate reductase iron-sulfur subunit |
| 4545-Rv | GCGTATTGCCGATGACATAAGG |  |
| 1413-Fw | TGTGCTTCCCGTTTATCTTCC | Malate dehydrogenase, Phosphate acetyltransferase |
| 1413-Rv | CTTCCCCTTCCGCCAGTA |  |
| 2526-Fw | CGTGAGTCTCCCGCCTGTA | Glucose-1-phosphatase, secreted |
| 2526-Rv | CCCGCCACTGTTGTTCTGT |  |
| 3256-Fw | CTCAACGGCAAACTGAAAGG | Glyceraldehyde-3-phosphate dehydrogenase, type I |
| 3256-Rv | CGTCTGGGTGGCATCAAAT |  |
| 4856-Fw | GCGTGATTATGGCGTTAGGTG | MIP family channel proteins |
| 4856-Rv | GAACAGCGGGACGAGGAAG |  |
| 4857-Fw | AAAGGCGGCACTCGTATTCC | Glycerol kinase, putative |
| 4857-Rv | GCAACCGATGGTGGTCAGC |  |
| 4858-Fw | CCGCTATGGGTCAGGCAAAT | Fructose-1,6-bisphosphatase, class II |
| 4858-Rv | TGGCATCGTGGCGAGGTT |  |
| 895-Fw | CCGCAACGCTTTCCATAA | Bacterial cellulose synthase subunit A (representing the concentration of bacteria) |
| 895-Rv | TCAGCCGCAAAGAAGTGAG |  |

**Table S2** Primers used in construction and determination of genetically engineered strain

| **Primers** | **Sequence (5'--3')** |
| --- | --- |
| Fpg1u | CCCAAGCTTCTGCAACCTGCCATGGAAT |
| Fpg1l | TCTTTCCGGCATTTAAACAGCTCGCACCGTAACAG |
| Fpg2u | CGAGCTGTTTAAATGCCGGAAAGAAGAGAAAACG |
| Fpg2l | CCGCTCGAGGGCAAAATCGGTCAAGATGATT |
| Fpgk1u | CGCCAGCCCATCCAGCAGTTTC |
| Fpgk1l | CCACCCCGGTGAACAGCTCCTC |
| Fpgk2u | CAACCACTACCTGAGCACCCAG |
| Fpgk2l | GGTGAGCGGCATTGGAGTGTAT |

**Table S3** The physical and chemical properties of the crude oil obtained from the Luliang Oilfield

| **Properties** | **Description** |
| --- | --- |
| Classification | General black oil |
| Density of ground crude oil | 0.881 g/cm^3^ |
| Viscosity of ground crude oil | 23.48 mPa•s |
| Wax content | 3.53% |
| Freezing point | -24℃ |
| Initial distillation point | 178℃ |
| Density of formation crude oil | 0.854 g/cm^3^ |
| Viscosity of formation crude oil | 20.46 mPa•s |
| Dissolved gas-oil ratio | 23 m^3^/m^3^ |
| Crude oil volume coefficient | 1.057 |
| Crude oil compression coefficient | 18.19 × 10^-4^/MPa. |
